# Disparities in Food Insecurity and Academic Achievement Among California Public University Students: An Intersectional Approach

**DOI:** 10.3390/nu16213728

**Published:** 2024-10-31

**Authors:** Sonali Singh, Erin E. Esaryk, Erika Meza, Tolani Britton, Suzanna M. Martinez

**Affiliations:** 1 Department of Epidemiology and Biostatistics, University of California, 550 16th St., 2nd Floor, San Francisco, CA 94158, USA; sonali.singh@ucsf.edu (S.S.); erin.esaryk@ucsf.edu (E.E.E.); 2 Center for Population Studies and Development Studies, Harvard University, 9 Bow St., Cambridge, MA 02138, USA; emeza@hsph.harvard.edu; 3 School of Education, University of California, 2121 Berkeley Way, 4th Floor, Berkeley, CA 94720, USA; tabritton@berkeley.edu

**Keywords:** food insecurity, higher education, college students, intersectionality

## Abstract

**Background/Objectives**: Historically racialized status (HRS) and low socioeconomic position (SEP) are independent risk factors for food insecurity and poor academic achievement among college students. Despite increased enrollment of students from historically racialized groups and low SEP, little is known regarding the intersectional experience of these contemporary student characteristics with food security status or academic achievement. The purpose of this study was to examine the intersections of racialized status and SEP with food insecurity and academic achievement among undergraduate students attending a public university system in California. **Methods**: This cross-sectional study included 1170 undergraduates who utilized their campus food pantry between June and August 2019 at nine University of California campuses. Racialized status and SEP were used to construct four distinct intersectional positions: (1) White, not low SEP (i.e., traditional students; reference), and three contemporary student groups: (2) White, low SEP; (3) HRS, not low SEP; and (4) HRS, low SEP. Using regression analyses, these intersectional positions were examined with food insecurity and grade point average (GPA), while controlling for other student characteristics. **Results**: HRS, low SEP students had significantly higher odds of experiencing food insecurity (OR = 2.72; 95% CI: 1.52–4.97) and lower GPA (B = −0.14, *p* = 0.05) than traditional students, after adjustment. **Conclusions**: Contemporary students are at increased risk of food insecurity and lower academic achievement compared to traditional students.

## 1. Introduction

The U.S. college student population has undergone a significant sociodemographic transformation over the last 40 years [1,2]. Historically, access to U.S. higher education was predominantly granted to White, middle- to upper-class individuals, many of whom were financially dependent on their parents and enrolled immediately after high school (referred to as “traditional” students by the U.S. Board of Education) [3,4]. The U.S. higher education system was designed with traditional students in consideration [5], potentially explaining why it inadequately serves the needs of the “contemporary” student population (also known as “nontraditional” students). Racial, ethnic, and socioeconomic diversity on campuses has grown considerably. In 2021, approximately 47% of undergraduates identified with a historically racialized group (defined as non-White racial groups within the sociohistorical context of the U.S.) compared to 17% in 1980 [2,6,7]. Additionally, 32% of undergraduates nationwide receive a Federal Pell Grant (an indicator of low-income status) [8], and 37% are the first generation in their families to enroll in postsecondary education [9]. The rising cost of attendance (i.e., tuition, fees, and cost of living) makes it challenging for contemporary students to meet their basic needs and thrive in higher education.

Despite food being an essential need, up to 40% of college students experience food insecurity (defined as the limited ability to acquire adequate and safe food due to a lack of money and other resources) [10,11], underscoring a significant public health challenge across U.S. college campuses. College students disproportionately experience food insecurity at a higher prevalence than U.S. households (13%) [10,11,12]. Research in the student population has found that food insecurity is linked to low fruit and vegetable intake, high body mass index, poor sleep quality, less physical activity, and poor mental and physical well-being [13,14,15]. Studies also suggest a negative relationship between food insecurity and academic achievement, including lower academic performance and graduation rates [13,15,16,17].

Various independent factors related to food insecurity and poor academic achievement have been identified among college students [18,19,20,21], many of which coincide with the increasingly common characteristics of contemporary students, such as identifying with a historically racialized group, low-income status, and first-generation student status [2,8,9]. Most studies on college food insecurity and academic achievement examine race and class as independent factors, also referred to as a single-axis approach. A limitation of the single-axis approach is that it fails to recognize how interlocking systems of power and oppression (i.e., racism, sexism, and classism), uniquely affect individuals across multiple interconnected identity factors to shape their life outcomes. An understanding of how interlocking systems of oppression impact the college experience for contemporary students is lacking.

Intersectionality is a framework to understand interlocking systems of oppression that is rooted in early writings of U.S. Black women—many with histories of enslavement [22,23]. The Combahee River Collective, a group of Black lesbian feminist activists, was the first to develop an integrated approach to understanding class relationships [24]. Coined by Kimberlé Crenshaw in 1989, intersectionality is used to understand the unique discrimination Black women in the U.S. faced base on multiple systems of power and privilege (i.e., the unique effects of racism and sexism) [25]. The framework offers a way to identify and address challenges due to multiple forms of inequality operating together, rather than through a single-axis framework (i.e., racism or sexism alone) [25,26]. Intersectionality seeks to highlight and capture forms of discrimination occurring at the juncture of interlocking systems of privilege and oppression [27]. The theory posits that experiences at these intersections of sociohistorical axes of oppression are not merely the sum effect of the two other systems but rather a unique experience specific to that intersection.

Historically racialized status (HRS) and low SEP are independently related to food insecurity and academic achievement among college students [18,20]. Given the interconnectedness of these factors via interlocking systems of oppression, it is critical to use an intersectional approach when examining their relationship with food insecurity and academic achievement among college students, both contemporary and traditional [28]. A 2017 study across the University of California system (*n* = 84,752) found that the prevalence of food insecurity among undergraduate students with low-income status alone (51%) was lower than among those at the intersection of low-income and first-generation student status (59%) and among students at the intersection of low-income, first-generation, and historically racialized status (e.g., African American/Black, Hispanic/Latino, American Indian; 63%) [29]. Additionally, in 2018, the 23-campus California State University system (*n* = 24,324) found that while 49% of first-generation students experienced food insecurity, its prevalence was considerably higher than average among Black first-generation students (69%) and lower than average among White first-generation students (47%) [30]. Applying an intersectional lens within the context of college food security and academic achievement allows for a more nuanced understanding of the health and academic disparities that contemporary students experience in higher education. Thus, the purpose of this study was to examine how the intersectional positions of racialized status and socioeconomic position relate to food insecurity and academic achievement among undergraduate students attending a large, public university system in California.

## 2. Materials and Methods

### 2.1. Context

This study took place within the context of the systemwide University of California (UC) Basic Needs Initiative, launched in 2015 to address food and housing insecurity among UC students and their impact on students’ well-being and academic performance [31].

### 2.2. Study Design

This cross-sectional study used data from the 2019 UC Basic Needs Survey and the UC Institutional Research and Academic Planning Office (IRAP). Between June and August 2019, undergraduate and graduate students who utilized campus basic needs services were recruited from Basic Needs Center listservs from all 10 UC campuses. The initial purpose of the survey was to examine campus basic needs resource utilization in relation to basic needs security. A Qualtrics survey was emailed to 35,537 students. Of those who received an email invitation, 1855 responded (5.2% response rate). Students provided informed consent before beginning the online survey, which included questions on financial circumstances and responsibilities, employment, general health, and food security. Student demographic information (e.g., age, sex, race and ethnicity, campus affiliation, student level, and grade point average [GPA]) was obtained through IRAP.

### 2.3. Participants

Of the 1855 participants who responded to the survey, graduate students were excluded (*n* = 326) due to limited financial aid information for this group. Of the 1529 undergraduate participants remaining in the sample, we excluded observations with missing data on first-generation status (*n* = 3), Pell Grant receipt (*n* = 1), racial and ethnic group (*n* = 36), financial circumstance (i.e., sending money to family, *n* = 4; financial stress, *n* = 2; ability to afford an emergency, *n* = 3; and food insecurity, *n* = 319). Thus, the final analytic sample included 1170 participants. Participants who had missing data were compared to participants with complete data, with no significant difference between groups in terms of age and sex.

### 2.4. Independent Variables

Racialized status was determined using participants’ self-reported race and ethnicity information, categorized as White, Black, Latino/a, Asian, Filipino/Pacific Islander, or American Native. Participants who identified as Black, Latino/a, Asian, Filipino/Pacific Islander or American Native were combined and coded as HRS (1) vs. White (0) [6].

For socioeconomic position (SEP), participants self-reported if they were the first generation in their family to attend college, whether they received a Pell Grant (a need-based federal grant provided to income-eligible undergraduates) [32], and whether they received Work Study (a federal program that provides employment opportunities to students in need of financial support to help pay for education expenses). Using these variables, a dichotomous composite variable was created by meaningful grouping to indicate low SEP. Participants who met the criteria of being a first-generation student or demonstrated financial need were coded as low SEP (1) and those who did not were coded as not low SEP (0) [33].

Racialized status and SEP were then used to create four mutually exclusive intersectional positions: (1) White, not low SEP (reference group), hereafter referred to as “traditional students”; (2) White, low SEP; (3) HRS, not low SEP; and (4) HRS, low SEP. In this study, we refer to the latter three intersectional positions as “contemporary students”.

### 2.5. Dependent Variables

Food security was assessed using the validated USDA 6-Item Short-Form Module [34] in reference to the most recent academic term (Spring 2019). The number of affirmative answers to the six questions was summed to create a raw score using the USDA’s coding scheme: food secure (score of 0–1), low food security (score of 2–4), and very low food security (score of 5–6). Low food security and very low food security were combined and coded as food insecure (1) vs. food secure (0).

Academic achievement was assessed using cumulative GPA (scale: 1.00–4.00) obtained through institutional records.

### 2.6. Covariates

Covariates included sex (male/female), age (years), and campus affiliation. We also controlled for financial circumstances [35,36], such as sending money to family (yes/no), experiencing stress due to personal finances more than half the time (yes/no), ability to afford an emergency expense of any amount (yes/no), daily frequency of fruit and vegetable intake (continuous) [37], and the number of depressive symptoms. Depressive symptoms were assessed based on the Patient Health Questionnaire-8 [38] and validated among college students, with a range of 0–8 (e.g., hopelessness, feeling overwhelmed, exhaustion, loneliness, sadness, overwhelming anxiety, overwhelming anger, and feeling so depressed it is difficult to function) [39].

### 2.7. Statistical Analyses

All data analyses were conducted in RStudio version 2023.06. Differences in characteristics by student group were tested using chi-square tests of independence for categorical variables and Kruskal–Wallis tests for continuous variables. Multiple logistic regression was applied to estimate the odds ratio (OR) of experiencing food insecurity for each contemporary student group compared to the traditional student group. A multiple linear regression model was also used to investigate the relationship between these intersectional positions and GPA. Covariates were tested in models and those with a *p*-value > 0.20 were excluded from the final models for parsimony. The final model examining food insecurity included age, sex, campus affiliation, sending money to family, experiencing stress due to personal finances more than half the time, ability to afford an emergency expense of any amount, and number of depressive symptoms. The final model examining GPA included age, sex, campus affiliation, sending money to family, ability to afford an emergency expense of any amount, and food security status. *p*-values were considered statistically significant if <0.05.

## 3. Results

Participant characteristics by intersectional position are reported in Table 1. On average, participants were 21.7 years old (SD = 3.4), predominantly female (78%), and Latino/a (39%) or Asian (35%). Over half (56%) of participants received a Pell Grant, 59% were first-generation college students, and 37% participated in federal Work Study. Approximately 62% of participants experienced food insecurity during their most recent academic term and a majority (66%) reported feeling frequent stress due to personal finances. The overall sample had a mean GPA of 3.13 (SD = 0.60).

HRS, low SEP participants made up the majority of the sample (68%), followed by HRS, not low SEP (17%); White, low SEP (8%); and traditional participants (7%). Among the four intersectional positions, food insecurity was most prevalent among White, low SEP participants (71%), followed by HRS, low SEP participants (69%). About 43% of HRS, low SEP participants reported sending money to family, compared to 17% of their traditional counterparts. HRS, low SEP participants also had the lowest GPA of the four groups, with a mean of 3.05 (SD = 0.60).

### 3.1. Intersectional Positions of Racialized Status and Socioeconomic Position in Relation to Food Insecurity 

HRS, low SEP participants (OR = 2.72, 95% CI: 1.52–4.97) and White, low SEP participants (OR = 2.55, 95% CI: 1.26–5.72) had higher odds of experiencing food insecurity compared to traditional participants, after controlling for covariates. No significant relationship was found between HRS, not low SEP participants and food insecurity (Table 2). 

### 3.2. Intersectional Positions of Racialized Status and Socioeconomic Position in Relation to GPA

HRS, low SEP participants had a significantly lower GPA compared to traditional participants after controlling for covariates (B = −0.14, *p* = 0.05; Table 3). The White, low SEP group and HRS, not low SEP group were not significantly related to GPA. Participants in the HRS, low SEP group had the lowest predicted mean GPA of 3.08 (95% CI: 3.04–3.12). The traditional and HRS, not low SEP participant groups had the same adjusted predicted GPA of 3.21 (95% CI: 3.08–3.34 and 3.13–3.29, respectively), and the White, low SEP participant group had the highest predicted GPA of 3.28 (95% CI: 3.17–3.40).

## 4. Discussion

This study examined the intersectional positions of racialized status and socioeconomic position (SEP) in relation to food insecurity and academic achievement among traditional and contemporary college students. The primary focus was on contemporary students marginalized by multiple systems of power and oppression, such as racism and classism. Among undergraduate students attending a large, public university system in California, we found that, regardless of racialized status, students with low socioeconomic position had an increased risk of food insecurity. We also found that students with historically racialized status (HRS) and low socioeconomic position were more likely to underperform academically compared to traditional students. These findings illustrate the challenges that contemporary college students with intersectional experiences face in their pursuit of higher education.

The current study found that students at the nexus of HRS and low SEP had approximately three times the odds of experiencing food insecurity compared to traditional participants. This finding aligns with a prior study among undergraduate and graduate students attending a university in West Virginia’s Appalachian region (*n* = 2653), which examined intersectional positions of race and first-generation status [40]. In this study by Olfert et al., Black first-generation students were four times more likely to experience food insecurity than White first-generation students [40]. While our study focused more broadly on historically racialized groups as a whole, both studies suggest that students with historical exclusion based on racialized status and socioeconomic position face barriers that contribute to food insecurity. These barriers may stem from longstanding systems of power and privilege in higher education (i.e., classism and racism) as well as social systems that contribute to students’ financial insecurity, which may impact their ability to meet their basic needs.

We also found that White, low SEP students were more likely than their traditional peers to experience food insecurity. This finding, combined with our results regarding the relationship between HRS, low SEP participants and food insecurity, suggests that socioeconomic position alone—based on income and parental education—is a significant social determinant of access to adequate, nutritious food [33]. Food assistance programs, such as the Supplemental Nutrition Assistance Program (SNAP) for low-income households and the National School Lunch Program for K-12, are some of the nation’s strongest efforts against food insecurity. While reducing food insecurity at the household level and in children are outlined in the Healthy People 2030 key objectives, college students remain underrepresented in these national priorities and continue to encounter barriers to accessing SNAP [20,41]. Recently, the Biden Administration’s National Strategy on Hunger, Nutrition and Health highlighted that “SNAP’s college student eligibility restrictions are out of date” given that contemporary students are more likely to have limited incomes [42]. Advocating for the elimination of SNAP student restrictions could address food insecurity among contemporary students with low SEP [43].

This current study also found that participants at the intersection of HRS and low SEP were more likely to underperform academically compared to traditional students, and, among the four intersectional position groups, had the lowest predicted GPA. Similarly, Whitcomb and colleagues analyzed 10 years of institutional data from a large public university in Pennsylvania (*n* = 24,567) and found comparable results when examining differences in yearly GPA among undergraduate students categorized by various combinations of historically excluded characteristics (i.e., gender, race and ethnicity, low-income, and first-generation status) [44]. The results revealed that students at the intersection of any “underrepresented minority” racial and ethnic group other than White or Asian, with low-income status, and first-generation status consistently had lower GPAs from their first year of college until graduation compared to White, not low-income, continuing generation students (i.e., traditional students) [44]. Furthermore, the study found that this student group had the lowest mean GPA among sixteen observed intersectional positions [44]. Despite differences in racial and ethnic categorization between Whitcomb et al.’s and this current study, both suggest that students who experience multiple systems of oppression are more susceptible to lower academic achievement than traditional students. Compared to their higher-income peers, students at the intersection of historically racialized status and low socioeconomic position face numerous barriers in higher education that impede academic success, including working longer hours to help pay for their basic needs, the greater mental cost of adapting to college culture, and the time spent seeking resources to succeed [45,46]. There is a critical need to remove unequitable barriers in higher education for contemporary students to thrive, as traditional students continue to benefit from systems of power and privilege embedded in the U.S. higher education system, which reinforce the opportunity gap.

One noteworthy finding was that the White, low SEP participant group had the highest predicted GPA (equivalent to a B+), compared to the other three intersectional positions (B equivalent). Similarly, Whitcomb and colleagues found that White and Asian college students with low socioeconomic position performed better academically than their peers who identified as an “underrepresented minority” and were not low income [44]. It is worth noting that students in this current study were recruited from campus food pantry listservs, and therefore likely experience barriers to food access. Also, fewer White, low SEP participants were in the first generation in their family to attend college (51%) compared to HRS, low SEP participants (81%). Despite economic hardship, White students may still benefit from systemic privileges that contribute to academic success. For example, compared to students from historically racialized groups, White students tend to experience stronger senses of belonging on campus, which is associated with increased engagement, increased confidence in academic abilities, and ultimately, improved academic achievement [47]. In contrast, students from historically racialized groups may have a more difficult time acclimating to college culture than their traditional peers as contemporary students are less likely to have attended secondary schools that provided preparation for college-level work, more likely to work additional hours to support themselves, and less likely to have generational college knowledge—all of which may impact their academic success [48,49,50,51]. The academic achievement gap between White students and students from historically racialized groups is well established throughout K-12 [52,53]. Historically, discriminatory housing practices, such as redlining, have resulted in segregated neighborhoods and school districts, ultimately impacting school funding which is connected to academic performance [54]. On average, U.S. public school districts serving predominantly racially and ethnically diverse students receive $2226 less per student than predominantly White districts [55]. Moreover, in California, high poverty, diverse school districts receive nearly $4000 less per student than high poverty, White school districts [55]. School funding directly impacts students’ access to technology, advanced coursework and college preparation, and can significantly impact long-term academic success [54]. Thus, the persistent achievement gap is a repercussion of long-term inequitable access to resources and opportunities rooted in systemic racism and historical exclusion, as supported by our study findings [53].

This study has several limitations. First, causality cannot be inferred due to the cross-sectional design. Another potential limitation of this study is the low survey response rate (5.2%). However, a 2017 study of college surveys found that a response rate of 5% to 10% was considered reliable, provided a sample size of at least 500 students [56]. Additionally, we utilized a sample of UC undergraduate students who accessed their campus food pantry; therefore, findings may not be generalizable to the overall UC undergraduate student population or other student populations. The small sample sizes of traditional and the White, low SEP students also require the results to be interpreted with caution. Multiple studies in the UC system have found that White students are less likely to struggle with meeting their basic needs compared to students from historically racialized groups, which may explain the small sample sizes of intersectional positions consisting of White students [18,57]. We were also unable to control for additional uncollected variables that could impact food security status or academic success in college, such as caring for dependents or academic preparation (i.e., high school GPA and standardized test scores) [21,58,59]. It is worth noting that not all students in this sample were experiencing food insecurity. In prior research in this sample, we have found that some students use the food pantry as a buffer to prevent them from experiencing food insecurity [60]. Lastly, small sample sizes did not allow for disaggregated racial and ethnic groups, given that the parent study was not based on the intersectionality framework. To advance social justice, future research could apply the intersectionality framework in study designs to examine unique intersections within race and ethnicity, given the heterogeneity of contemporary students. Despite these limitations, the nine campuses included comprised several Hispanic-Serving Institutions and spanned both rural and urban regions in California. This study provides significant contributions by applying an intersectional lens to evaluate associations between food insecurity and academic achievement among a diverse population within a large, public university system in California.

## 5. Conclusions

This study examined disparities in food insecurity and academic achievement among contemporary and traditional college students by accounting for intersectional positions of racialized status and socioeconomic position. Findings suggest that systemic racism and structural barriers may contribute to contemporary students’ ability to afford and access nutritious food and to thrive academically in higher education, particularly for historically racialized students with low socioeconomic position. Study findings also suggest that, while food insecurity is mainly driven by socioeconomic position, the underlying causes of food insecurity and academic performance disparities may be deeply rooted in societal systems where privilege and oppression operate. Future research involving college student populations should examine the impact of historically racialized status and low socioeconomic position on health outcomes and degree completion. Additionally, policymakers should prioritize reducing financial barriers for contemporary students by increasing federal financial aid, such as the Pell Grant, to ensure they can meet their basic needs and succeed academically.

## Figures and Tables

**Table 1:** Characteristics of undergraduate student participants at nine University of California campuses, by intersectional positions of racialized status and socioeconomic position (*n* = 1170).

			Contemporary Students			
Characteristic	Total (*n* = 1170)	Traditional (*n* = 78)	White, Low SEP (*n* = 95)	HRS, Not Low SEP (*n* = 201)	HRS, Low SEP (*n* = 796)	*p*
Sex, *n* (%)						0.921
Female	914 (78.1)	60 (76.9)	72 (75.8)	159 (79.1)	623 (78.3)	-
Male	256 (21.9)	18 (23.1)	23 (24.2)	42 (20.9)	173 (21.7)	-
Age, mean ± SD	21.7 ± 3.4	21.0 ± 1.2	22.9 ± 5.2	20.9 ± 1.4	21.8 ± 3.5	**<0.001**
First-generation, *n* (%)	692 (59.1)	0 (0.0)	48 (50.5)	0 (0.0)	644 (80.9)	**<0.001**
Pell Grant recipient, *n* (%)	649 (55.5)	0 (0.0)	74 (77.9)	0 (0.0)	575 (72.2)	**<0.001**
Work study participant, *n* (%)	430 (36.8)	0 (0.0)	43 (45.3)	0 (0.0)	387 (48.6)	**<0.001**
Race and ethnicity, *n* (%)						**<0.001**
American Native	18 (1.5)	0 (0.0)	0 (0.0)	5 (2.5)	13 (1.6)	-
Asian	406 (34.7)	0 (0.0)	0 (0.0)	133 (66.2)	273 (34.3)	-
Black	51 (4.4)	0 (0.0)	0 (0.0)	9 (4.5)	42 (5.3)	-
Filipino/Pacific Islander	67 (5.7)	0 (0.0)	0 (0.0)	23 (11.4)	44 (5.5)	-
Latino/a	455 (38.9)	0 (0.0)	0 (0.0)	31 (15.4)	424 (53.3)	-
White	173 (14.8)	78 (100.0)	95 (100.0)	0 (0.0)	0 (0.0)	-
Live off-campus, *n* (%)	793 (68.3)	61 (78.2)	70 (73.7)	138 (69.7)	524 (66.3)	0.094
Frequent stress due to personal finances, *n* (%)	772 (66.0)	39 (50.0)	75 (78.9)	86 (42.8)	572 (71.9)	**<0.001**
Sends money to family, *n* (%)	447 (38.2)	13 (16.7)	35 (36.8)	59 (29.4)	340 (42.7)	**<0.001**
Unable to afford emergency expense of any amount, *n* (%)	286 (24.4)	11 (14.1)	22 (23.2)	16 (8.0)	237 (29.8)	**<0.001**
Depressive symptoms, mean ± SD	2.9 ± 2.5	2.7 ± 2.5	3.3 ± 2.4	2.4 ± 2.4	3.0 ± 2.5	**0.008**
Daily frequency of fruit and vegetable intake, mean ± SD	4.4 (2.3)	4.5 (2.2)	4.4 (2.1)	4.3 (2.2)	4.4 (2.4)	0.927
Food secure, *n* (%)	440 (37.6)	50 (64.1)	28 (29.5)	115 (57.2)	247 (31.0)	**<0.001**
Food insecure, *n* (%)	730 (62.4)	28 (35.9)	67 (70.5)	86 (42.8)	549 (69.0)	**<0.001**
Low food security	429 (36.7)	18 (23.1)	39 (41.1)	59 (29.4)	313 (39.3)	-
Very low food security	301 (25.7)	10 (12.8)	28 (29.5)	27 (13.4)	236 (29.6)	-
GPA, mean ± SD	3.13 ± 0.60	3.32 ± 0.52	3.28 ± 0.58	3.28 ± 0.58	3.05 ± 0.60	**<0.001**

Bold values represent statistical significance at *p* < 0.05. Abbreviations: HRS = Historically racialized status (defined here as American Native, Asian, Black, Filipino/Pacific Islander, or Latino/a.); SEP = Socioeconomic position; GPA = Grade point average.

**Table 2:** Associations between intersectional positions of racialized status and socioeconomic position with food insecurity among University of California undergraduate student participants (*n* = 1170).

		Food Insecurity	
Variable	OR	95% CI	*p*
Intersectional position (Ref: Traditional)	-	-	-
White, low SEP	2.55	1.26–5.72	**0.011**
HRS, not low SEP	1.71	0.90–3.32	0.105
HRS, low SEP	2.72	1.52–4.97	**0.001**
Regularly sends money to family	1.49	1.09–2.04	**0.012**
Frequently stressed due to personal finances	5.49	4.04–7.50	**<0.001**
Unable to afford an emergency expense of any amount	3.25	2.18–4.95	**<0.001**
Depressive symptoms	1.18	1.11–1.26	**<0.001**
Regularly sends money to family	1.49	1.09–2.04	**0.012**

Bold values represent statistical significance at *p* < 0.05. Abbreviations: OR = Odds Ratio; CI = Confidence Interval; HRS = Historically racialized status; SEP = Socioeconomic position. Model adjusted for age, sex, and campus affiliation.

**Table 3:** Associations between intersectional positions of racialized status and socioeconomic position with GPA among University of California undergraduate student participants (*n* = 1170).

		GPA	
Variable	Stand β	95% CI	*p*
Intersectional position (Ref: Traditional)	-	-	-
White, low SEP	0.07	−0.11–0.24	0.441
HRS, not low SEP	−0.01	−0.16–0.14	0.945
HRS, low SEP	−0.14	−0.27–0.00	0.048 *
Regularly sends money to family	−0.09	−0.16–−0.02	**0.012**
Unable to afford an emergency expense of any amount	−0.17	−0.25–−0.09	**<0.001**
Food insecure	−0.18	−0.25–−0.10	**<0.001**

Bold values represent statistical significance at *p* < 0.05. One asterisk indicates marginal significance at *p* < 0.05. Abbreviations: HRS = Historically racialized status (defined here as students who identify as American Native, Asian, Black, Filipino/Pacific Islander, or Latino/a.); SEP = Socioeconomic position; GPA = Grade point average. Model adjusted for age, sex, and campus affiliation. * *p* < 0.05.

## Data Availability

The data presented in this study are available on request from the corresponding author due to privacy reasons.
